# Epidemiology, Patients’ Journey and Healthcare Costs in Early-Stage Non-Small-Cell Lung Carcinoma: A Real-World Evidence Analysis in Italy

**DOI:** 10.3390/ph16030363

**Published:** 2023-02-27

**Authors:** Diego Luigi Cortinovis, Valentina Perrone, Elisa Giacomini, Diego Sangiorgi, Margherita Andretta, Fausto Bartolini, Giuseppe Taurino, Marco Belfiore, Emilia Sicari, Luca Degli Esposti

**Affiliations:** 1 Division of Oncology, Department of Medicine and Surgery, San Gerardo Hospital, University of Milano-Bicocca, 20126 Monza, Italy; 2 CliCon S.r.l. Società Benefit, 40137 Bologna, Italy; 3 Unità Operativa Complessa, Assistenza Farmaceutica Territoriale, Azienda Unità Sanitaria Locale Socio Sanitaria 8 Berica, 36100 Vicenza, Italy; 4 Pharmaceutical Department, Unità Sanitaria Locale Umbria 2, 05100 Terni, Italy; 5 Pharmaceutical Department, Unità Sanitaria Locale Toscana Nord Ovest, 56121 Pisa, Italy; 6 Roche Spa, 20900 Monza, Italy

**Keywords:** real-world evidence, healthcare costs, eNSCLC cancer

## Abstract

This real-world analysis aims to estimate the epidemiology and economic burden related to early-stage non-small-cell lung carcinoma (eNSCLC) in the clinical practice Italian setting. An observational analysis was performed using administrative databases linked to pathological anatomy data, covering around 2.5 mln health-assisted individuals. From 2015 to mid-2021, eNSCLC patients staged II–IIIA treated with chemotherapy after surgery were included. Patients were stratified into those presenting loco-regional or metastatic recurrence during follow-up and annualized healthcare direct costs covered by the Italian National Health System (INHS) were estimated. In 2019–2020, the prevalence of eNSCLC was 104.3–117.1/million health-assisted subjects, and the annual incidence was 38.6–30.3/million. Data projected to the Italian population estimated 6206 (2019) and 6967 (2020) prevalent and 2297 (2019) and 1803 (2020) incident cases. Overall, 458 eNSCLC patients were included. Of them, 52.4% of patients had a recurrence (5% loco-regional-recurrence, 47.4% metastatic-recurrence). Healthcare total direct costs/patient averaged EUR 23,607, in particular, in the first year after recurrence, costs averaged EUR 22,493 and EUR 29,337 in loco-regional and metastatic-recurrence patients, respectively. This analysis showed that about one-half of eNSCLC patients stage II–IIIA experience a recurrence, and in recurrence patients, total direct costs were almost two-fold those of no-recurrence patients. These data highlighted an unmet clinical need, as the therapeutic optimization of patients at early stages.

## 1. Introduction

Lung cancer remains a scientific and healthcare topic of great interest, representing the most frequent and lethal cancer worldwide [1]. Non-small-cell lung cancer (NSCLC) is the predominant form of lung cancer, with about one-third of the cases diagnosed with Stage I–III disease [2]. In spite of the advances in diagnostic procedures and the availability of current therapeutic options, the management of NSCLC still remains an open challenge for researchers and clinicians, above all, for the high frequency of relapse, which involves up to 60% of patients [3].

In this context, the problem of recurrent metastasis in NSCLC patients is crucial, as it causes detrimental rebounds not only on patients themselves and neoplasm prognosis, but also on caregivers, the healthcare system and society [4]. Unfortunately, despite the improved outcomes of metastatic NSCLC over the last decades, resulting from the introduction of combination chemotherapy, small molecules targeting mutant proteins, 5-year survival of these patients remains low, about 26% in stage IIIB, 10% in stage IVA and less than 1% in stage IVB patients [5].

Hence, it is important to identify patients with early NSCLC since timely treatment might result in higher chances of improving prognosis and survival [6].

While surgery alone represents the standard of care in stage I, patients with stage II–III NSCLC often need adjuvant treatment with platinum-based chemotherapy or radiotherapy after surgery; in selected stage II–III NSCLC, also a neo-adjuvant treatment can be performed after the multidisciplinary discussion [7]. On the other hand, the management of metastatic NSCLC is complicated and leads to elevated healthcare costs, mainly related to the necessity of cancer-related hospitalizations and long-term therapy [6].

Thus, this real-world evidence (RWE) analysis was undertaken to: (i) estimate the epidemiology of early-stage non-small-cell lung carcinoma (eNSCLC) treated with chemotherapy after surgery, (ii) assess the management of these patients, (iii) determine the impact of disease relapse and metastasis recurrence on patients’ outcomes and (iv) estimate the economic burden associated with the journey of these patients in a clinical practice setting in Italy.

## 2. Results

**Figure 1:**
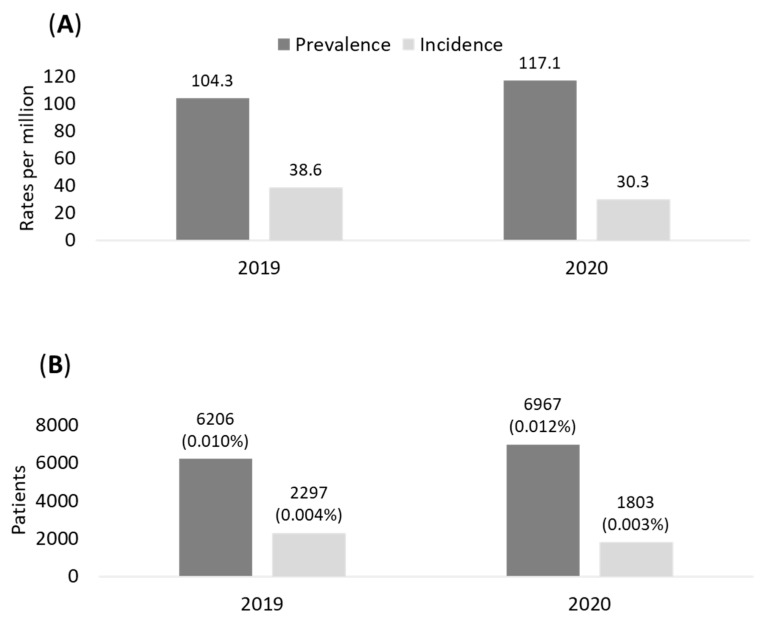
Prevalence and incidence of eNSCLC patients in stage II–IIIA with chemotherapy after surgery during 2019–2020 in the sample of population (**A**) and projection on Italian population (**B**).

The prevalence and incidence of eNSCLC patients treated with chemotherapy after surgery during 2019 and 2020 in the sample population analyzed are shown in Figure 1A. The epidemiology evaluations refer to the time of surgery as index date. The prevalence of stage II–IIIA NSCLC patients in our population was 104/million health-assisted subjects in 2019 and 117/million health-assisted individuals in 2020. The incidence was 39 and 30/million individuals in 2019 and in 2020, respectively (Figure 1A). Data projected to the entire Italian population estimated 6026 and 6967 prevalent cases in 2019 and 2020, respectively; in addition, 2297 and 1803 incident cases were estimated among the Italian population during 2019 and 2020, respectively (Figure 1B).

**Figure 2:**
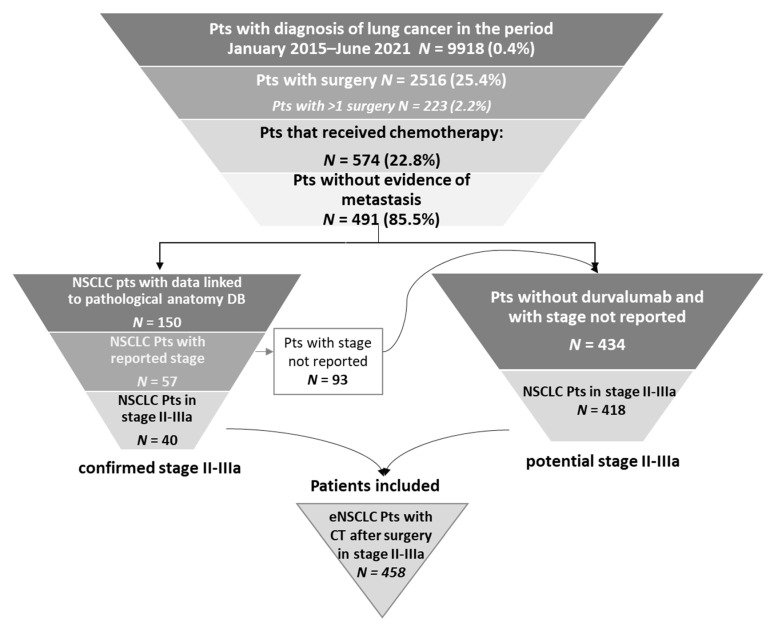
Sequential steps of the selection of NSCLC patients treated with chemotherapy after surgery in stage II–IIIA. Abbreviations: CT, chemotherapy; NSCLC, non-small-cell lung cancer; Pts, patients.

Between January 2015 and June 2021, 9918 patients with lung cancer diagnoses were identified, 2516 (25.4%) of whom had undergone surgery (Figure 2). Specifically, 1692 (67.2%) had lobectomy, 188 (7.5%) pneumonectomy and 636 (25.3%) other types of surgical lung resection. Furthermore, 2.2% (223/9918) underwent more than one surgery (for such patients, first surgery was considered as the index date). Among the 2516 patients with surgery, 574 (22.8%) received chemotherapy. Specifically, 491 of the 574 chemotherapy-treated patients were without evidence of metastasis: 40 of them were confirmed as being in stage II–IIIA by pathological anatomy database and 418 were identified as potential stage II–IIIA since they did not present metastasis or durvalumab-based treatment. Thus, a total of 458 patients were included in the pool of eNSCLC patients in stage II–IIIA treated with chemotherapy after surgery and evaluated for the present analysis (Figure 2).

The baseline demographic and clinical characteristics of the selected 458 eNSCLC patients are described in Table 1. The patients averaged 67.4 years (median age: 69 years), and 60.5% were males (as expected). The comorbidity profile assessed by the Charlson index showed that about 42% had a low level of comorbidity (Charlson index < 1), 40% had a mild level of comorbidity (Charlson index = 1) and the remaining 18% had a moderate to severe comorbidity profile (Charlson index ≥ 2).

As Table 2 shows, over half of the patients included in the analysis had recurrence (52.4%) within a mean time of 8.9 ± 10.6 months: specifically, 5% had a loco-regional recurrence, while 47.4% experienced a metastatic recurrence (of which 27.9% distant recurrence and 19.4% unspecified recurrence).

**Figure 3:**
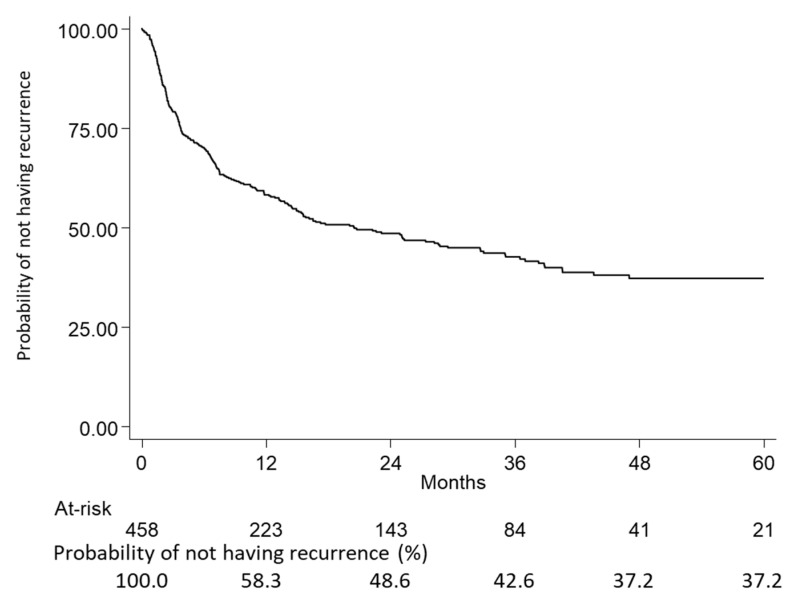
Kaplan–Meier curves showing the probability of not having recurrence at 5 years in the overall population made up of eNSCLC patients with chemotherapy after surgery in stage II–IIIA (study period January 2015–June 2021).

Within 5 years (60 months) from inclusion, the probability of not having recurrence was estimated by Kaplan–Meier curves; 52.4% of patients had a recurrence after a median time of 20.5 months. Moreover, 46.9% of included patients had a recurrence within 24 months, and this share remained almost stable up to 60 months (52.4%), reaching a plateau state from 48 to 60 months (Figure 3 and Table 3 and Table 4).

In a sub-group of patients (*N* = 40) with available stage reported in the pathological anatomy database, the recurrence rate was compared between stage II and stage IIIA. As shown in Supplementary Table S1, there was a lower trend of frequency of overall recurrences (which were metastatic recurrence in all patients) in stage II than in stage IIIA patients (55.6% vs. 63.6%, respectively); specifically, distant recurrence accounted for 44% and 36% of stage II and stage IIIA patients, respectively. In addition, recurrences had a median earlier onset in stage II patients compared to stage IIIA (overall: 6.3 vs. 11.0 months; distant metastatic: 12.4 vs. 13.0 months). Details on the number and time interval for loco-regional recurrences are not reported for the distinct tumor stages due to the scarcity of pathological anatomy data. The demographic and clinical characteristics of the progression of patients stratified according to the type of recurrence are detailed in Supplementary Table S2. The mean age of the 23 patients with loco-regional recurrence was 67 years (median 70 years), while among the 217 patients with metastatic recurrence, the mean age was 67.8 (median 69 years). Males tended to be more represented in the metastatic group (62.2%) than in the loco-regional group (34.8%). However, these data comparing the patients’ characteristics for the two groups should be interpreted with caution in view of the small sample size, especially for the loco-regional recurrent patient group.

As shown in Supplementary Figure S1A, among the 217 patients experiencing metastatic recurrence, the most frequent metastasis sites were liver (13.4%), bone (21.7%), brain (22.1%), followed by other respiratory organs (34.1%) and lymph nodes (40.6%). The number of metastatic sites was 1 in 72 (33.2%), 2 in 47 (21.7%), 32 in 39 (18.0%), above 3 in 14 patients (6.5%) and not reported in 45 cases (20.7%) (Supplementary Table S3). Among patients who experienced more than one recurrent metastasis, the most frequent combinations of the various metastatic sites are described in Supplementary Figure S1B: lung/lymph nodes 7.8%, brain/lymph nodes 3.2%, brain/lung 2.3%, bone/lymph nodes 2.3%, bone/lung 1.8%, bone/liver/lymph nodes 1.8%, adrenal gland/lymph nodes 1.8%, liver/lymph nodes 1.8%, brain/lung/lymph nodes 1.8% and liver/lung/lymph nodes 1.8%.

**Figure 4:**
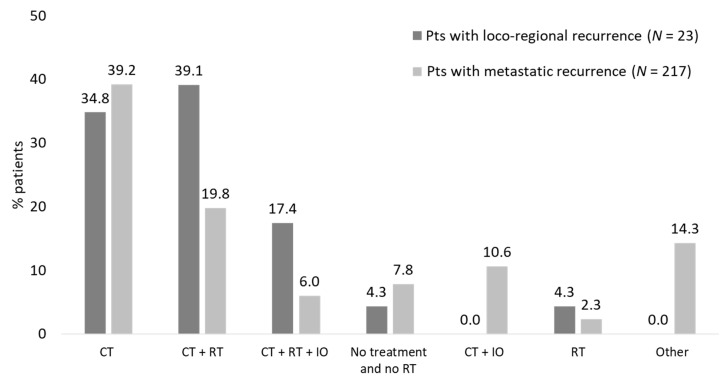
Therapeutic patterns after recurrence patients with eNSCLC in stage II–IIIA treated with chemotherapy after surgery divided according to the type of recurrence (study period January 2015–June 2021).

The therapeutic patterns after recurrence show that aspecific chemotherapy alone or plus radiotherapy were more frequently observed in patients with loco-regional occurrence. Nevertheless, the difference in treatment patterns may be affected by the methodology applied since the presence of specific schemes such as immunotherapy/targeted therapies was used to identify metastatic recurrence (Figure 4).

**Figure 5:**
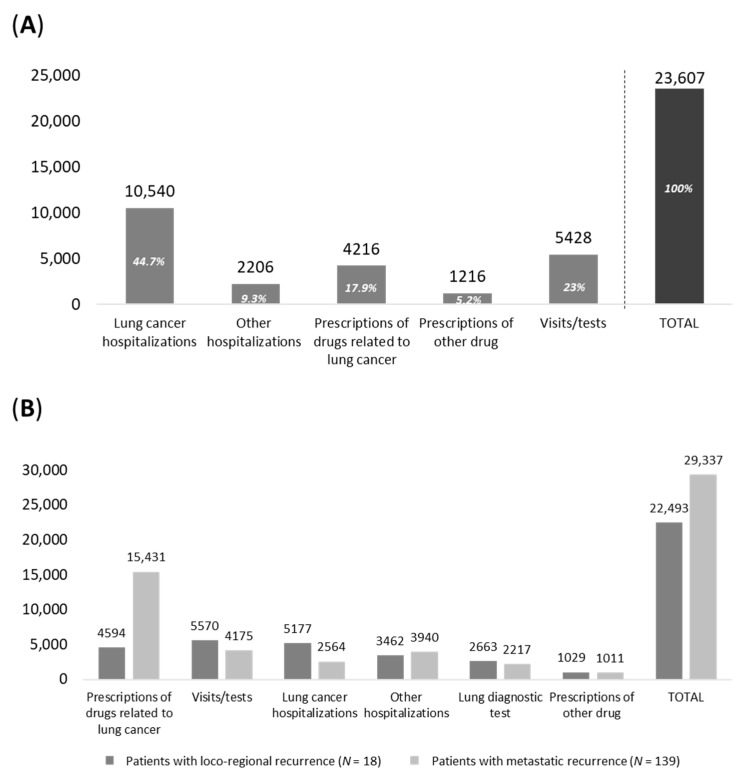
Healthcare costs during (**A**) the first year of follow-up among alive eNSCLC patients who were treated with chemotherapy after surgery in stage II–IIIA (study period January 2015–June 2019) and (**B**) during first year after recurrence among alive patients—study period January 2015–June 2020.

The analysis of healthcare resource use and relative cost was evaluated in the included patients. As shown in Figure 5A, during the first year of follow-up (after surgery), the mean annual cost per patient averaged out to EUR 23,607, mainly driven by lung cancer-related hospitalization costs (EUR 10,540, 45% of total cost), expenditures related to lung cancer medications (EUR 4216, 18%) and specialist diagnostic visits/tests/exams (EUR 5428, 23%). When considering patients with loco-regional or metastatic recurrence during the first year after recurrence, the mean healthcare costs were EUR 22,493 and 29,337, respectively (Figure 5B), highlighting an almost 30% increase in overall costs among the latter. Such a difference is to be mainly attributed to the expenditure for lung cancer treatments, which is approximately 2.5 times higher in patients with metastatic recurrence (on average, EUR 4594 in patients with loco-regional recurrence and EUR 15,431 (+236%) in those with metastatic recurrence), while all the other expenditure items associated with those patients are lower than in the loco-regional patient subgroup.

To describe time-dependent healthcare consumption, resource use and costs were evaluated in overall eNSCLC patients treated with chemotherapy after surgery, during the first three years of follow-up. As reported in Supplementary Table S4, during the second and third years, patients receiving lung cancer-related medications witnessed a decreasing trend, while no variations were observed in relation to the consumption of other drugs, as well as in costs related to tests/visits (including PET/CT and CT scan). Moreover, patients undergoing lung cancer-related hospitalization decreased significantly over time. This trend also mirrored that of healthcare direct costs. In fact, we observed an almost 80% decrease in lung cancer-related hospitalization costs throughout the years (EUR 10,243 during the first year and EUR 671 and 694 during the second and third years) (Supplementary Table S5); conversely, costs related to lung cancer medications witnessed an increasing trend (almost 22%) in the second/third years.

In addition, an analysis was performed focusing on the annualized healthcare resource use and related costs during a larger time-horizon of 2 years after surgery among alive patients, based on recurrence status. As shown by Table 5, a trend towards a rise in resource consumption was observed in patients with recurrence (especially those experiencing a metastatic recurrence).

**Figure 6:**
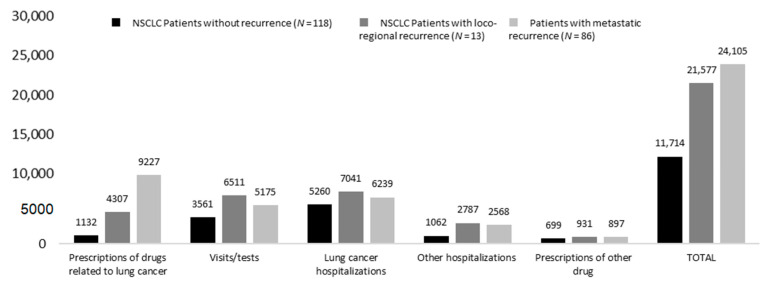
Annualized healthcare direct costs during first 2 years after surgery among alive patients—inclusion period January 2015–June 2019, stratified by recurrence status.

Likewise, as described in Figure 6, the mean annualized costs at 2 years from surgery were almost two-times higher in patients with metastatic and loco-regional recurrence (EUR 24,105 and 21,577, respectively) compared to those with no recurrence (EUR 11,714). This difference was mainly driven by expenditures related to lung cancer drugs (which increased by almost 80% in patients with metastatic recurrence versus those without recurrence) and by visits/tests.

## 3. Discussion

The present RWE analysis carried out among a sample of 2.5 mln patients assisted by three Italian LHUs provides insights into the epidemiology of stage II–IIIA eNSCLC patients treated with chemotherapy after surgery, their clinical-therapeutic journey with a focus on the impact of disease recurrence as well as on the healthcare economic burden covered by the NHS in a setting of clinical practice.

Performed between January 2015 and June 2021, the analysis evaluated the number of NSCLC patients in stage II–IIIA treated with chemotherapy after surgery; by projecting these data to the entire Italian population at a yearly level (2019 and 2020), we estimated a prevalence rate of 0.1% (approximately 6500 patients) with an incidence rate of 0.004% (approximately 2000 new diagnoses per year). Consistent with Italian and international data [8,9,10], eNSCLC patients analyzed were relatively elderly with a median age of 69 years, predominantly male and with a significant proportion of comorbidities, as about 60% of the patients had a Charlson index equal or above 1. Concerning the clinical course of the disease, about one-half of the patients experienced a recurrence (52.4%) after a median time of 20.5 months; 47% of cases manifested a metastatic recurrence, while the remaining 5% of patients experienced a loco-regional recurrence. Our data are consistent with previous reports on the pattern of postoperative recurrence risk in eNSCLC, indicating a rate of distant metastasis between 14.0% and 23.0%, and loco-regional recurrence around 5.0% [11]. Instead, Uramoto et al. reported that 17%, 44% and 39% of the recurrences were confined to local, distant and both sites, respectively [12]. The possible underestimation of loco-regional recurrence found in the present analysis can be explained by the diagnostic proxy used for its identification. Among patients with metastatic recurrence, the most frequent metastatic site was lymph nodes (41%), followed by other respiratory organs (34%) and brain or bones (22%), as previously reported [13].

Data on time to relapse estimated through Kaplan–Meier curves confirmed a bi-modal trend in the relapse occurrence, with a rapid peak recorded at about 9 months after surgery, and a second onset registered in almost 47% of patients after two years from surgery. Five years after the recurrence, 37.2% of patients can be considered recovered. This finding is in agreement with the fact that 30% to 55% of patients with NSCLC develop recurrence and die of their disease despite curative resection [11,12].

The analysis of therapeutic approaches used after recurrence, considering the availability of different therapeutic approaches during the analysis period, indicated that aspecific chemotherapy and radiotherapy were more commonly prescribed in patients with loco-regional occurrence. Furthermore, we found that almost 23% of patients received adjuvant chemotherapy after surgery; such a percentage is lower than the one reported by an observational analysis carried out in the US, in which the share of patients who received any adjuvant chemotherapy was 57% [14,15]. This difference could be attributable to the traceability of chemotherapy within the database used in the current analysis. In a recent Italian real-world study, the percentage reached around 40% (89/223 patients with surgery), which, however, has been reported among patients with stage III, such that more advanced patients could have been included [7].

Analysis of the consumption of healthcare resources and costs (in terms of pharmacological treatments, diagnostic tests, specialist visits and hospital admissions) of eNSCLC patients treated with chemotherapy after surgery in stage II–IIIA averaged almost EUR 23,600, driven mainly by lung cancer-related hospitalization costs and expenditure-related to lung cancer medications, such as, for instance, the tyrosine kinase inhibitors, which radically changed the management of patients with eNSCLC [16]. In addition, patients with metastatic recurrence received a greater amount of prescriptions for lung cancer drugs than patients experiencing a loco-regional recurrence, and their management resulted in higher costs for the Italian National Health Service (INHS), mainly due to cancer-related hospitalizations and specialist visits and tests. Thus, lung cancer hospitalization was confirmed to be the most impactful expenditure item. It is important to notice that the expenses for patients with loco-regional recurrence were always lower compared to patients with metastatic recurrence, regardless of the cost. These expected results could be explained by the complex management of metastatic patients in terms of the type of drug, number of administrations and number of lines of therapy. High-cost treatments are often used in metastatic subjects, while in loco-regional relapses, chemotherapy and radiotherapy are more widespread.

These findings must take into account some limitations, mainly lying in the retrospective observational study design and data extraction from administrative databases. Region/LHUs administrative databases have progressively improved the quality of the collected data. Nevertheless, some information may be missing; if the necessary information was missing for a given patient, that patient was excluded from the analysis. Our cohort of patients reflected real clinical practice by evaluating data from a subset of health-assisted individuals. A possible flaw to consider is the scarcity of clinical data available in the administrative databases on comorbidities and other potential confounders that could have influenced the present results. Since the comorbidities herein analyzed were addressed based on any available data before inclusion (using a proxy of diagnosis), there might be incomplete capture of these variables among patients that might have affected results. A further limitation is represented by the lack of data regarding adverse reactions experienced by these patients: their occurrence could represent not only a source of discomfort for patients (and, by consequence, a possible area of improvement for patient management) but also an additional cost for the INHS. The analyses were carried out only in eNSCLC patients treated with chemotherapy after surgery, thus not considering those without chemotherapy medication. The absence of metastasis detection could be slightly overestimated since it was proxied by absence of hospitalization diagnosis or specific treatments.

The analyses on patients stratified between those with loco-regional versus metastatic recurrence (in terms of recurrence assessment, treatment patterns and costs) could have been affected by the small sample size of subgroups. Moreover, the methodology applied to determine the difference in treatment patterns among subgroups might have impacted the results, such as the presence of specific schemes, e.g., immunotherapy/targeted therapies, was used as a proxy to identify metastatic recurrence. Lastly, the epidemiological analysis was carried out with the aim to estimate the incidence and prevalence of eNSCLC patients treated with chemotherapy after surgery in stage II–IIIA; thus, it cannot be generalized to overall eNSCLC patients. Moreover, the prevalence estimation did not take into account the number of cancer survivor patients since these data are not retrievable in the administrative database. For the identification of patients on stage II–IIIA, those treated with chemotherapy were selected; this could have caused an underestimation of patients due to methodological limitations on the traceability of chemotherapy within the database and to the fact that patients without chemotherapy have not been considered. Ultimately, the absence of a validation of the algorithm must be acknowledged. Despite these limitations and the fact that administrative claims data are not collected with the purpose of supporting research, analysis of real-world data from large datasets can deliver key information about patient management in clinical practice settings by using “in-house” information already stored by the LHUs [17].

## 4. Materials and Methods

### 4.1. Study Design and Data Source

An observational retrospective analysis was conducted by integrating the administrative databases and pathological anatomy database of three Local Health Units (LHUs), including data of 2.5 million health-assisted individuals. Administrative databases collect information on healthcare resource costs covered by the INHS, and they allow for tracking health mobility, i.e., the resources provided outside the territorial LHU to each health-assisted subject.

The following databases were browsed: (i) beneficiaries’ database, which holds all demographic data for patients in analysis; pharmaceuticals database, which records the data on drug supplies for patients through anatomical therapeutic code (ATC), prescription date, number of packages; (ii) hospitalization database, which encloses hospitalization-related data for patients, namely date of hospitalization, main and secondary diagnosis identified by International Classification of Diseases, Ninth Revision, Clinical Modification (ICD-9-CM) and Diagnosis Related Group (DRG); (iii) outpatient specialist service database, i.e., laboratory tests and specialist visits database, which contains all data about diagnostic tests and visits for patients in the analysis; (iv) payment exemption database, which contains data of the exemption codes that avoid the contribution charge for services/treatments when specific diseases are diagnosed. The data collected from the administrative databases were linked with those retrieved from the pathological anatomy database, which contains the histopathological examination data of the patients included in the analyses.

For the current study, Italian Entities (representative of Italian population) were selected by their geographical distribution, data completeness, and the high-quality linked datasets. To guarantee patient privacy, each health-assisted subject by the LHUs included in the analysis was identified by an anonymous univocal numeric code (Patient ID) in order to make the electronic linkage between databases possible. This Patient ID code guarantees the anonymity of the extracted data in full compliance with UE Data Privacy Regulation 2016/679 (“GDPR”) and Italian D.lgs. n. 196/2003, as amended by D.lgs. n. 101/2018. All the results were produced in aggregated form, and they could not be connected to individual patients either directly or indirectly. The analysis was notified and approved by the local Ethics Committees of the Healthcare Departments involved in the study: Comitato Etico Regionale dell’Umbria (protocol number 19414/20/ON, approval date 16 September 2020); Comitato Etico per le Sperimentazioni Cliniche (CESC) della Provincia di Vicenza (Prot. N 1627, 28 October 2020); Comitato Etico Regionale per la Sperimentazione Clinica della Regione Toscana (protocol number 20190211, approval date 12 September 2019).

### 4.2. Study Population

From January 2015 to June 2021, eNSCLC patients (stage II–IIIA) were identified using an algorithm composed of sequential steps. First, adult patients (≥18 years) with available data during the whole study period diagnosed with lung cancer were recognized by ICD-9-CM codes 162.2, 162.3, 162.4, 162.5, 162.8, 162.9 (main or secondary diagnosis). Secondly, those who underwent surgery for eNSCLC (lobectomy, pneumonectomy or other lung resections (including local excision of the lesion or tissue of the bronchus, ICD-9-CM code 32.0 or other bronchus resection ICD-9-CM code 32.1 or local excision of lung injury or tissue, ICD-9-CM code 32.2) 90 days before the diagnosis date or at any time after diagnosis date were identified by surgery codes 32 (main or secondary). The index date corresponded to the time of the surgery. Third, in order to improve the tracking of eNSCLC and to narrow it down to stages II–III, based on the national guidelines available on patients’ inclusion period, the algorithm detected patients receiving chemotherapy during the 4-month period following the surgery date, by DRG code 410, or hospital/ambulatory procedures 99.25, 99.28, 99.29 or ATC code L01. The follow-up period began at the index date and ended at the end of the study period, patient’s death, or exiting the database (whatever occurred first).

In order to exclude patients in stages IIIB–C and IV and restrict the analysis to stages (resectable) II–IIIA only, patients with metastasis before the surgery date (identified by the ICD-9-CM codes 197–198) or by the prescription of immunotherapy/target therapy, were excluded. In particular, the following exclusion criteria were used to enhance the probability of removing patients with stages IIIB-C or lung cancer neuroendocrine phenotypes from the analysis: (i) absence of prescription for durvalumab (ATC L01XC28, L01FF03) during all available period before the index date; (ii) absence of prescription for etoposide (ATC code L01CB01), lanreotide (ATC code H01CB03) or octreotide (ATC code H01CB02) from diagnosis date to follow up [18].

Lastly, another selection was made based on the NSCLC morphology-based confirmed diagnosis of stage II–IIIA, by means of administrative database and pathological anatomy database linking, using the staging system available at patients’ inclusion.

For included patients (eNSCLC patients stage II–IIIA treated with chemotherapy after surgery), the period comprising the 12 months prior to the index date, was used to characterize patients (characterization period).

### 4.3. Epidemiology

The epidemiological analysis evaluated the prevalence and incidence of the study population during 2019 and 2020. In each year, prevalence was reported as number of alive cases per health-assisted subjects, and incidence as number of new cases per health-assisted subjects. The data were re-proportioned to the overall Italian population to estimate the prevalence and incidence of patients diagnosed with NSCLC in stage II–IIIa treated with chemotherapy after surgery.

### 4.4. Study Variables

Baseline demographic and clinical characteristics were collected for all patients, together with the type of metastasis recurrence and healthcare resource consumptions and costs associated with each patient.

#### 4.4.1. Demographic and Clinical Characteristics

Age was computed at baseline and presented as mean ± standard deviation (SD) and median; gender was reported as proportion of males.

Clinic characteristics were assessed using an updated version of the Charlson Comorbidity Index [19], namely a recently updated and expanded ICD-9 scoring system of the Charlson Comorbidity Index [20]. This system assigns a score (1 to 6) to each concomitant disease assessed in the previous 12 months based on drug treatment and hospitalizations; therefore, untreated/non-hospitalized comorbidities were not captured. Cancer was excluded from the score counting. The comorbidity index was reported as mean ± SD and as proportion of patients in each score.

#### 4.4.2. Loco-Regional and Metastatic Recurrence

In order to discriminate between loco-regional and metastatic recurrence, the following definition was applied based on the first detection of ICD-9-CM code/drugs during follow-up: (a) loco-regional recurrence was identified by the presence of ICD-9-CM code 196.1 “Secondary and unspecified malignant neoplasm of intrathoracic lymph nodes” or by the presence of a prescription of durvalumab; (b) metastatic recurrence was identified by distant metastasis (ICD-9-CM codes 196–198 excluding 196.1), or unspecified metastasis recurrence recorded in patients receiving immunotherapy or target therapy for advanced/metastatic status. The pattern of distant metastasis with the corresponding ICD-9-CM codes is described in Supplementary Table S6.

#### 4.4.3. Treatment Pathways

The treatments in analysis, including chemotherapy, immunotherapy, target therapy and radiotherapy were identified during all data periods available for the study, and all details are reported in Supplementary Table S7.

### 4.5. Evaluation of Healthcare Resource Consumption

Healthcare resource use and related costs in Euros (EUR, €) were evaluated during the first year of follow-up after surgery among the included alive patients. The estimation was focused on: (a) drug treatments related to lung cancer management (all treatments reported in Supplementary Table S7) and other comedications: (b) all-cause hospital admissions related to lung cancer and other causes; (c) all-cause outpatient specialized services, namely laboratory tests and specialist visits. Cost analysis was carried out for all the included patients and for the subgroups of those with loco-regional and/or metastatic recurrence. Furthermore, healthcare cost evaluation was assessed during a longer time horizon by considering the first two years of follow-up.

### 4.6. Statistical Analysis

All the analyses performed are descriptive. Continuous variables were presented as mean ± SD and median values, while categorical variables were expressed as numbers and percentages. Time to recurrence (calculated from index date to loco-regional or metastatic recurrence) was evaluated by using Kaplan–Meier curves. Patients without relapse were censored at the date of database availability or death.

For cost analysis, the costs of the outliers were identified as values exceeding the mean value three times the SD were excluded from the analysis. Following the “Opinion 05/2014 on Anonymization Techniques” drafted by the “European Commission Article 29 Working Party”, the analyses involving ≤3 patients were not reported (NR) for data privacy, as they were potentially traceable to single individuals. All analyses were performed using STATA SE version 17.0 (StataCorp LLC, College Station, TX, USA).

## 5. Conclusions

The present RWE analysis, which presents, based on our knowledge, the first evidence among Italian clinical setting, provides insights into the epidemiology of stage II–IIIA eNSCLC patients treated with chemotherapy after surgery, the role of disease recurrence and the rebounds on healthcare economic burden covered by the INHS. In the current analysis, about 52.4% of patients had a recurrence, resulting in a remarkable increase in INHS total direct costs: in fact, in patients with recurrence, total direct healthcare costs were almost two-fold higher than those of no-recurrence patients. Consequently, this analysis indicates that there is an unmet clinical need and room for improvement in the management of eNSCLC patients.

## Figures and Tables

**Table 1:** Demographic and clinical characteristics of eNSCLC patients who had chemotherapy after surgery in stage II–III (study period January 2015–June 2021).

	Overall Patients (*N* = 458)
Age, years, mean (SD)	67.4 (±8.4)
Age, years, median	69
Age categories (by 10 years)	
18–39, *n* (%)	NR
40–59, *n* (%)	80 (17.5%)
60–69, *n* (%)	168 (36.7%)
70–79, *n* (%)	185 (40.4%)
80–89, *n* (%)	23 (5.0%)
Male gender, *n* (%)	277 (60.5%)
Charlson index (score), mean (SD)	0.8 (±0.9)
Charlson index (categories)	
<1, *n* (%)	190 (41.5%)
1, *n* (%)	185 (40.4%)
2+, *n* (%)	83 (18.1%)

**Table 2:** Recurrence and time to recurrence in eNSCLC patients with chemotherapy after surgery in stage II–IIIA (study period January 2015–June 2021).

	Overall Patients (*N* = 458)
Patients with recurrence, *n* (%)	240 (52.4%)
Months to recurrence, mean (±SD)	8.9 (±10.6)
Months to recurrence, median [min–max]	4.1 [0.3–43.6]
Patients with loco-regional, *n* (%) recurrence	23 (5.0%)
Months to loco-regional recurrence, mean (SD)	5.9 (±5.8)
Months to recurrence, median [min–max]	3.3 [1.0–20.5]
Patients with metastatic recurrence, *n* (%)	217 (47.4%)
Patients with distant recurrence, *n* (%)	128 (27.9%)
Months to distant recurrence, mean (SD)	9.8 (±11.0)
Months to recurrence, median [min–max]	6.1 [0.3–43.6]
Patients with unspecified recurrence, *n* (%)	89 (19.4%)
Months to unspecified recurrence, mean (SD)	8.4 (±10.8)
Months to recurrence, median [min–max]	3.6 [0.1–67.1]

Continuous variables are presented as mean ± standard deviation (SD), categorical variables as numbers and percentages in brackets.

**Table 3:** Patients with recurrence and median time to recurrence.

**Median, months (95% CI)**	20.5 (14.6–29.5)
**Censored patients**	218 (47.6)
**Patients with recurrence**	240 (52.4)

**Table 4:** Probability of not having a recurrence.

Months	% Survival	95% CI	% pt Recurrence (on All Patients)
0	1.000		0 (0.0)
12	0.583	0.534–0.628	181 (39.5)
24	0.486	0.436–0.534	215 (46.9)
36	0.426	0.374–0.477	230 (50.2)
48	0.372	0.316–0.428	239 (52.2)
60	0.372	0.316–0.428	240 (52.4)

**Table 5:** Annualized healthcare resource use during the first 2 years after surgery among alive eNSCLC patients who were treated with chemotherapy after surgery in stage II–IIIA (inclusion period January 2015–June 2019).

Healthcare Resource Use	NSCLC Patients without Recurrence (*N* = 118)		Patients with Loco-Regional Recurrence (*N* = 13)		Patients with Metastatic Recurrence (*N* = 86)	
	Mean ± SD	*N* (%)	Mean ± SD	*N* (%)	Mean ± SD	*N* (%)
Drug prescriptions for lung cancer	1.9 (±3.0)	51 (43.2%)	2.8 (±3.9)	7 (53.8%)	4.3 (±4.4)	60 (69.8%)
Other drug prescriptions	10.9 (±4.3)	118 (100%)	16.4 (±5.6)	13 (100%)	12.8 (±5.4)	86 (100%)
Test	15.3 (±8.7)	118 (100%)	23.2 (±14.5)	13 (100%)	20.2 (±12.6)	86 (100%)
Visits	7.3 (±4.1)	117 (99.2%)	9.5 (±5.8)	13 (100%)	9.6 (±4.8)	86 (100%)
Lung cancer hospitalizations	0.7 (±0.6)	116 (98.3%)	1.2 (±0.7)	13 (100%)	1.0 (±0.9)	86 (100%)
Other hospitalizations	0.4 (±0.5)	51 (43.2%)	0.8 (±1.2)	10 (76.9%)	0.5 (±0.7)	53 (61.6%)

## Data Availability

Data are contained within the article and supplementary materials.

## Supplementary Materials

The following supporting information can be downloaded at: https://www.mdpi.com/article/10.3390/ph16030363/s1: Table S1. Recurrence and time to recurrence in eNSCLC patients who received chemotherapy after surgery in stage II–IIIA (study period January 2015–June 2021); Table S2. Demographic and clinical characteristics of NSCLC patients treated with chemotherapy after surgery in stage II–IIIA with recurrence (study period January 2015–June 2021); Table S3. Distribution of patients with metastatic recurrence based on the number of metastatic sites; Table S4. Healthcare resource use during first 3 years of follow-up among alive eNSCLC patients treated with CT after surgery in stage II–IIIA—study period January 2015–June 2018; Table S5. Healthcare resource direct costs during first 3 years of follow-up among alive eNSCLC patients with CT after surgery in stage II–IIIA—study period January 2015–June 2018; Table S6. Type of recurrent metastases evaluated with the related ICD-9-CM codes; Table S7. Treatments in analysis; Figure S1. (A) Proportion of eNSCLC patients treated with chemotherapy after surgery in stage II–IIIA experiencing metastatic recurrence according to the different sites (study period January 2015–June 2021). (B) Among patients who experienced more than one recurrent metastasis, the proportion of combinations of the various metastatic sites were reported.
