# Supplementary material for: Epidemiology, Patients’ Journey and Healthcare Costs in Early-Stage Non-Small-Cell Lung Carcinoma: A Real-World Evidence Analysis in Italy
Source: Pharmaceuticals (Basel). 2023 Feb 27;16(3):363. doi: 10.3390/ph16030363 (PMC10056991; doi:10.3390/ph16030363)
Supplement: Supplementary file 1 [file pharmaceuticals-16-00363-s001.zip › pharmaceuticals-2229823-supplementary.pdf]

Supplementary Materials

Table S1. Recurrence and time to recurrence in eNSCLC patients who received chemotherapy after surgery in stage II-IIIA (study period 01/2015–06/2021).

	Stage II	Stage IIIA
**Patients with recurrence, %**	55.6%	63.6%
Months to recurrence, mean (±SD)	11.8 (± 10.8)	16.1 (± 18.0)
Months to recurrence, median [min-max]	6.3 [1.5–29.5]	11.0 [0.6–67.1]
**Patients with loco-regional recurrence, %**	0.0%	0.0%
Months to loco-regional recurrence, mean (±SD)	/	/
Months to recurrence, median [min-max]	/	/
**Patients with metastatic recurrence, %**	55.6%	63.6%
**Patients with distant recurrence, %**	44.4%	36.4%
Months to distant recurrence, mean (±SD)	12.4 (± 12.0)	13.0 (± 13.1)
Months to recurrence, median [min-max]	6.2 [1.5–29.5]	5.0 [0.6–38.3]
**Patients with unspecified recurrence, %**	11.1%	27.3%
Months to unspecified recurrence, mean (±SD)	9.3 (± 4.3)	20.3 (± 23.8)
Months to recurrence, median [min-max]	6.3 [6.3–12.3]	14.1 [0.8–67.1]

Note: Calculated only for patients with available stage reported in Pathological Anatomy Database (N = 40): hence, data should be interpreted with caution in view of the smaller sample size of the subgroups.

Table S2. Demographic and clinical characteristics of NSCLC patients treated with chemotherapy after surgery in stage II-IIIA with recurrence (study period 01/2015─06/2021). Continuous variables are presented as mean ± SD, categorical variables as numbers and percentages in brackets.

	**Patients with loco-regional recurrence (*n* = 23)**	**Patients with metastatic recurrence (*n* = 217)**
Age, years, mean (±SD)	67.0 (± 8.3)	67.8 (± 8.5)
Age categories (by 10 years)		
18-39, n (%)	0 (0%)	NR
40-59, n (%)	6 (26.1%)	37 (17.1%)
60-69, n (%)	3 (13.0%)	74 (34.1%)
70-79, n (%)	13 (56.5%)	92 (42.4%)
80-89, n (%)	NR	13 (6.0%)
Male gender, n (%)	8 (34.8%)	135 (62.2%)
Glasheen index (score), mean (± SD)	0.9 (± 0.8)	0.8 (± 0.9)
Glasheen index (categories)		
<1, n (%)	8 (34.8%)	101 (46.5%)
1, n (%)	10 (43.5%)	80 (36.9%)
2+, n (%)	5 (21.7%)	36 (16.6%)

Table S3. Distribution of patients with metastatic recurrence based on the number of metastatic sites

**Number of metastatic sites**	**Patients, *n* (%)**
not reported in the DB (*n*, %)	45 (20.7%)
1 (*n*, %)	72 (33.2%)
2 (*n*, %)	47 (21.7%)
3 (*n*, %)	39 (18.0%)
≥4 (*n*, %)	14 (6.5%)

Table S4. Healthcare resource use during first 3 years of follow-up among alive eNSCLC patients treated with CT after surgery in stage II-IIIA - study period 01/2015-06/2018.

	**One-year follow-up**		**Two-years follow-up**		**Three-years follow-up**	
	Mean (±SD)	*N* (%)	Mean (±SD)	*N* (%)	Mean (±SD)	*N* (%)
Prescriptions of drugs related to lung cancer	2.8 (±4.2)	56 (41.2%)	1.6 (±3.8)	28 (20.6%)	2.5 (±5.7)	35 (25.7%)
Prescriptions of other drugs	15.2 (±6.0)	136 (100%)	8.9 (±5.1)	135 (99.3%)	9.1 (±6.0)	133 (97.8%)
Test	22.2 (±15.7)	136 (100%)	11.1 (±10.1)	134 (98.5%)	13.1 (±12.4)	133 (97.8%)
Visits	9.2 (±5.9)	133 (97.8%)	6.7 (±5.3)	133 (97.8%)	6.5 (±6.3)	129 (94.9%)
Fibrobronchoscopy	0.2 (±0.5)	20 (14.7%)	NI	NI	NI	NI
PET / CT and CT scan	3.9 (±1.7)	132 (97.1%)	2.6 (±1.5)	126 (92.6%)	2.4 (±1.6)	124 (91.2%)
Nuclear magnetic resonance	0.0 (±0.0)	0 (0%)	0.0 (±0.0)	0 (0%)	NI	NI
Lung cancer hospitalizations	1.5 (±1.0)	134 (98.5%)	0.1 (±0.4)	13 (9.6%)	0.1 (±0.5)	14 (10.3%)
Other hospitalizations	0.7 (±1.1)	61 (44.9%)	0.3 (±0.8)	27 (19.9%)	0.4 (±1.0)	30 (22.1%)

Table S5. Healthcare resource direct costs during first 3 years of follow-up among alive eNSCLC patients with CT after surgery in stage II-IIIA - study period 01/2015–06/2018

**NSCLC Patients with CT after surgery in stage II-IIIa (*N*=136)**	
	**Cost per patient (€)**
**One-year follow-up**	
Prescriptions of drugs related to lung cancer, mean (±SD)	2,842.6 (±10,323.7)
Prescriptions of other drugs, mean (±SD)	853.2 (±698.5)
Visits/tests, mean (±SD)	5,192.8 (±4128.1)
Lung cancer hospitalizations, mean (±SD)	10,243.0 (±3393.7)
Other hospitalizations, mean (±SD)	2,741.9 (±4787.3)
TOTAL	*21,873.4 (±12,960.2)*
**Two-years follow-up**	
Prescriptions of drugs related to lung cancer, mean (±SD)	3,749.4 (±10,471.6)
Prescriptions of other drugs, mean (±SD)	683.6 (±819.2)
Visits/tests, mean (±SD)	3,062.5 (±3949.7)
Lung cancer hospitalizations, mean (±SD)	671.1 (±2645.5)
Other hospitalizations, mean (±SD)	1,099.1 (±3465.0)
TOTAL	*9,265.7 (±12,831.5)*
**Three-years follow-up**	
Prescriptions of drugs related to lung cancer, mean, SD)	3,661.1 (±10,692.5)
Prescriptions of other drugs, mean (±SD)	745.6 (±1205.9)
Visits/tests, mean (±SD)	2,993.5 (±4528.8)
Lung cancer hospitalizations, mean (±SD)	693.6 (±2758.3)
Other hospitalizations, mean (±SD)	1,910.5 (±5543.3)
TOTAL	*10,004.2 (±14,059.6)*

Table S6. Type of recurrent metastases evaluated with the related ICD-9-CM codes.

**Type of metastases**	**ICD-9-CM codes**
Brain metastasis	198.3
Bone metastasis	198.5
Liver metastasis	197.7
Other respiratory organs metastasis	197.0, 197.1, 197.2, 197.3
Adrenal gland metastasis	198.7
Peritoneum metastasis	197.6
Lymph nodes metastasis	196 (excluded 196.1)
Other metastasis	197, 198 not considered above

Table S7. Treatments in analysis

Treatment	Identification codes
Aspecific chemotherapy	DRG code: 410 OR hospital/ambulatory procedures 99.25, 99.28, 99.29 OR ATC L01 excluded the treatments listed below
Pemetrexed	ATC code L01BA04
Nivolumab	ATC code L01XC17
Durvalumab	ATC code L01XC28
Atezolizumab	ATC code L01XC32
Pembrolizumab	ATC code L01XC18
Gefitinib	ATC code L01XE02/L01EB01
Erlotinib	ATC code L01XE03/L01EB02
Afatinib	ATC code L01XE13/L01EB03
Crizotinib	ATC code L01XE16/L01ED01
Ceritinib	ATC code L01XE28/L01ED02
Osimertinib	ATC code L01XE35/L01EB04
Alectinib	ATC code L01XE36/L01ED03
Dabrafinib + Trametinib	ATC code L01XE23/L01EC02 + L01XE25/L01EE01
Radiotherapy	(ICD9 V580 or procedure 922, 923)

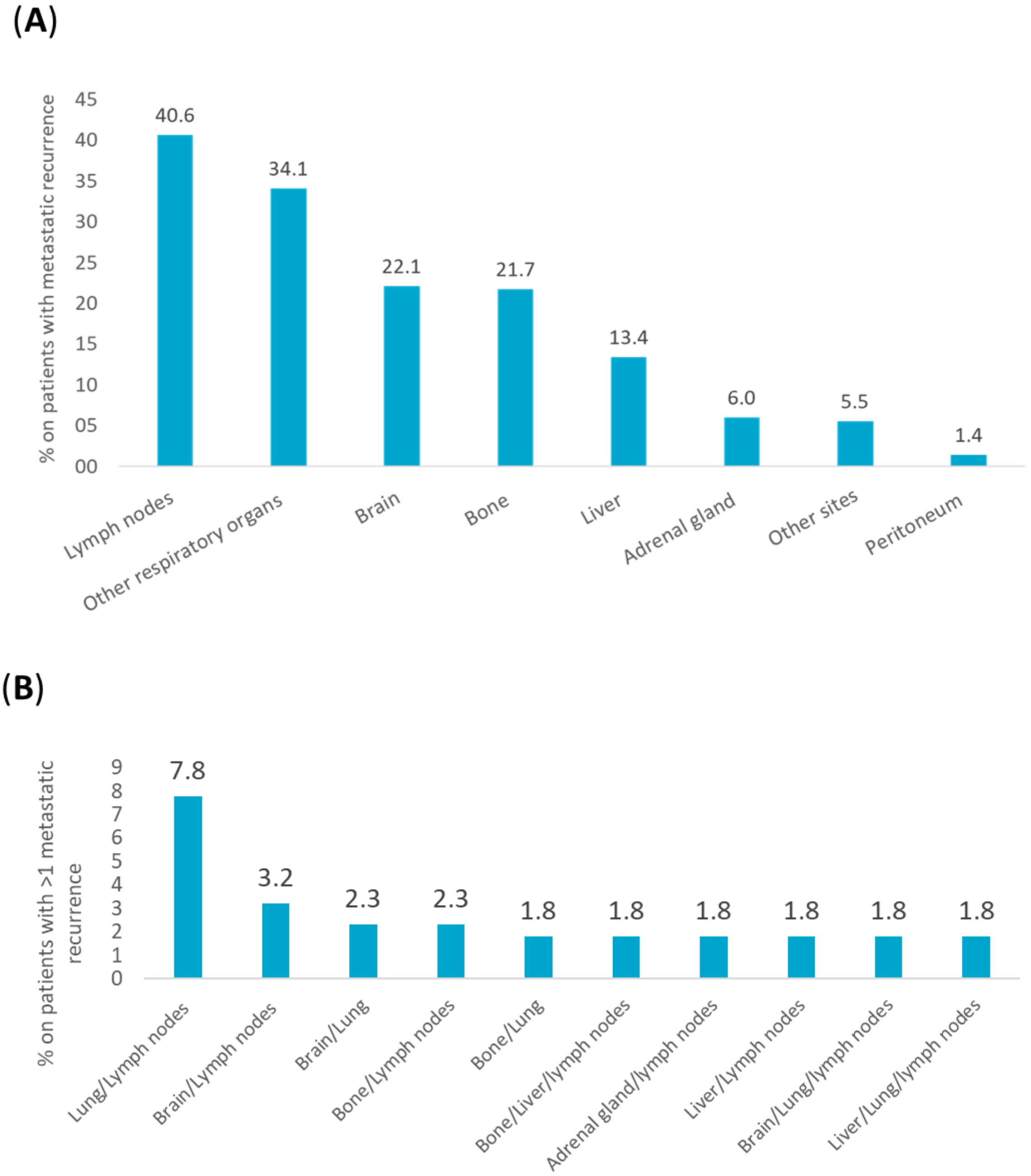

Figure S1. (A) Proportion of eNSCLC patients treated with chemotherapy after surgery in stage II- IIIA experiencing metastatic recurrence according to the different sites (study period 01/2015-06/2021). **(B)** Among patient who experienced more than one recurrent metastasis, the proportion of combinations of the various metastatic sites were reported.
